# Striking tick-borne virus diversity and potential reservoirs documented during One-Health-based cross-sectional screening in Anatolia

**DOI:** 10.1186/s13071-025-07046-w

**Published:** 2025-10-10

**Authors:** Ender Dinçer, Mehmet Özkan Timurkan, Bekir Oğuz, Emre Ozan, Nüvit Coşkun, Şemistan Kızıltepe, Pelin F. Polat Dinçer, Adem Şahan, Deniz Yalçınkaya, Ömer Faruk Gökçeçik, Mehmet Berat Nayir, Sercan Hüseyin Bayendur, Brian Patrick Bourke, Yvonne-Marie Linton, Koray Ergunay

**Affiliations:** 1 Department of Virology, Faculty of Veterinary Medicine, Dokuz Eylül University, Izmir, Türkiye; 2 Department of Virology, Faculty of Veterinary Medicine, Atatürk University, Yakutiye, Erzurum, Türkiye; 3 Department of Parasitology, Faculty of Veterinary Medicine, Van Yüzüncü Yıl University, Van, Türkiye; 4 Department of Veterinary Experimental Animals, Faculty of Veterinary Medicine, Ondokuz Mayıs University, Samsun, Türkiye; 5 Department of Virology, Faculty of Veterinary Medicine, Kafkas University, Kars, Türkiye; 6 Tuzluca Vocational School, Iğdır Üniversitesi, Iğdır, Türkiye; 7 Department of Internal Medicine, Faculty of Veterinary Medicine, Dokuz Eylül University, Izmir, Türkiye; 8 Department of Internal Medicine, Faculty of Veterinary Medicine, Harran University, Şanlıurfa, Türkiye; 9 Department of Medical Services, Vocational School of Health Services, Toros University, Mersin, Türkiye; 10 Veterinary Control Institute Directorates, Ministry of Agriculture and Forestry, Bornova Veterinary Control Institute, İzmir, Türkiye; 11 Department of Virology, Faculty of Veterinary Medicine, Burdur Mehmet Akıf Ersoy University, Burdur, Türkiye; 12 Department of Internal Medicine, Faculty of Veterinary Medicine, Afyon Kocatepe University, Afyon, Türkiye; 13 Department of Entomology, Smithsonian Institution–National Museum of Natural History (NMNH), Washington, DC USA; 14 Smithsonian Museum Support Center, MRC-534, 4210 Silver Hill Road, Suitland, MD 20746-2863 USA

**Keywords:** Tick, Pathogen, Virus, Anatolia, CCHFV, Tamdy

## Abstract

**Background:**

An expansion of recently described human pathogenic tick-borne viruses from Central Asia toward Europe has been documented. Located on important bird migration routes, Anatolia is an intercontinental crossing hub with various climactic zones and with an abundance of endemic tick species. We sought to investigate tick-borne viruses utilizing a One Health approach encompassing host-removed ticks and host samples.

**Methods:**

We collected host-attached ticks and accompanying plasma in 2023–2024 at locations in 20 provinces representing the 7 distinct geographical regions in Anatolia. The hosts comprised cattle, sheep, dogs, goats, and tortoises. The ticks were morphologically identified, processed in pools, and these pools, along with plasma from cattle, sheep and goats, were subjected to nucleic acid purification and complementary DNA synthesis. Viruses were screened by generic (nairovirus) and specific (Jingmen tick virus, JMTV; Tacheng tick virus 1, TcTV-1; Tacheng tick virus 2, TcTV-2; and Tamdy virus, TAMV) amplification assays and characterized by sequencing.

**Results:**

A total of 93 animal plasma samples and 1265 samples from 11 tick species were screened in 192 pools. Crimean–Congo hemorrhagic fever virus (CCHFV) was detected in five tick species in ten pools (5.2%). Three distinct virus lineages, including Europe 1 and 2, as well as Africa 1, were noted. TcTV-1 was identified in 6 tick species in 12 pools (6.3%) and in a cattle plasma sample. Analysis of the nucleoprotein-encoding sequences revealed two separate virus clades, distinct from those reported from Asia and Europe. TAMV was identified in two tick species (1%). We further detected JMTV in 7 pools (3.6%), with sequences forming a new clade phylogenetically closer to viruses of Asian origin than local strains. Finally, highly divergent sequences of a novel nairovirus, forming a distinct group sharing ancestors with TcTV-1, TAMV, and pangolin/tick-associated nairoviruses, was observed in four pools (2%), comprising *Haemaphysalis parva* ticks.

**Conclusions:**

We described a previously undocumented diversity of tick-borne viral pathogens, CCHFV, TcTV-1, and JMTV, in Anatolia. Possible animal reservoirs of TcTV-1 were identified. These pathogens and TAMV should be considered in the diagnostic workup of cases with symptoms associated with tick bites and in future surveillance efforts.

**Graphical Abstract:**

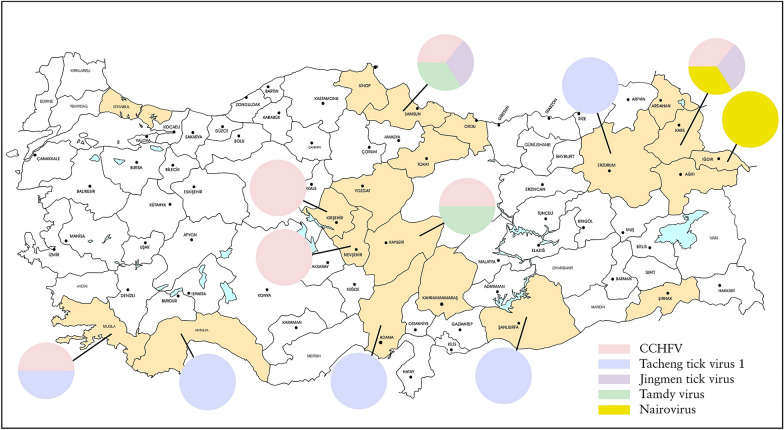

**Supplementary Information:**

The online version contains supplementary material available at 10.1186/s13071-025-07046-w.

## Background

Ticks (order Ixodida) are obligate blood-feeding ectoparasites of mammals, birds, and reptiles, acting as primary biological vectors of viral pathogens affecting livestock, companion animals, and wildlife [1]. The prevalence and spectrum of tick-borne viruses has been on the rise for the several decades and has become a global public health threat, in addition to causing economic losses due to affected livestock and wildlife morbidity. Diseases due to tick-borne viruses are further impacted by climate, globalization, population movements, and modifications of landscapes and natural habitats [2]. Moreover, the capacity of ticks to transmit pathogens across multiple life stages generates complex epidemiological networks and opportunities for spillover to susceptible hosts and reservoirs [3].

A diverse group of viruses circulating between ticks and vertebrate hosts have been recognized as causative agents of human or animal infections. They are taxonomically classified into a single DNA (*Asfarviridae*) and several RNA virus families [1]. The majority of tick-borne viral pathogens with significant public health impact possess RNA genomes and are classified in the families *Nairoviridae* and *Flaviviridae* [4]. Members of the *Nairoviridae* family, such as Crimean–Congo hemorrhagic fever virus (CCHFV), typically have tri-segmented negative-sense RNA genomes, where open reading frames (ORFs) on each segment encode a nucleoprotein (segment S), a glycoprotein precursor (segment M), and an RNA-directed RNA polymerase (RdRP) (segment L) [5]. Flaviviruses, exemplified by tick-borne encephalitis virus (TBEV), possess a positive-sense RNA genome that contains a single long ORF, which is translated into a polyprotein that requires protease cleavage to generate mature viral proteins [6]. In addition to the observed expansion and increased incidence of these major tick-borne viral agents of public concern, several newly-described viruses have been documented as causing mild-to-severe human infections following tick-borne transmission [1]. One such group includes Jingmen tick virus (JMTV) and Alongshan virus, which comprise a multicomponent genome composed of four segments, two of which encode viral proteins genetically and functionally related to flaviviruses [7]. Other recently described tick-borne pathogenic nairoviruses, such as Tacheng tick virus 1 (TcTV-1) and phenuiviruses such as Tacheng tick virus 2 (TcTV-2), have further been documented as expanding from their regions of initial detection [8]. Although severe symptoms of central nervous system or hemorrhagic disease might occur in affected humans or animals, many tick-borne viral infections are mild and only manifest as undifferentiated febrile disease, which may pass unnoticed or misdiagnosed [2]. Therefore, screening is crucial to reveal circulating and newly introduced pathogens and implement accurate diagnostics and mitigation strategies.

Located between Asia, Europe, and the Middle East, Türkiye occupies the Anatolian lands of Asia Minor and Eastern Thrace region of the Balkan Peninsula. With regions of diverse ecological features, Anatolia provides suitable habitats for many tick species and maintains a natural transmission zone for vector-borne infections between Asia, Africa, and Europe [9]. The primary tick-borne virus circulating in Anatolia is CCHFV, which emerged in 2002 with symptomatic cases annually reported and prior evidence for human exposure [10]. Moreover, other viruses including JMTV have recently been identified in ticks from various parts of Anatolia, with unexplored human or animal health impact [10]. In this study, we sought to investigate tick-borne viruses using a One Health approach encompassing host-removed ticks and host samples, utilizing a broad range of amplification assays and sequencing to characterize viral genomes.

## Methods

### Sample collection

**Figure 1:**
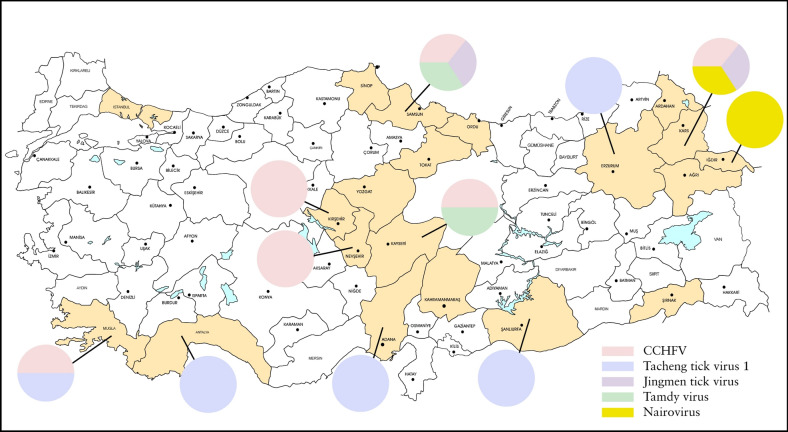
Illustrative map of the sampling provinces and viruses detected. Viruses are depicted by distinct colors and pie charts do not represent quantitative data

The tick specimens were collected during May–October in 2023–2024 at locations in 20 provinces representing the 7 distinct geographical regions, comprising Istanbul (Marmara); Mugla (Aegean); Adana, Antalya, Kahramanmaras (Mediterranean); Kayseri, Kirsehir, Nevsehir, Yozgat (Central Anatolia); Ordu, Samsun, Sinop, Tokat (Black Sea); Agri, Ardahan, Erzurum, Igdir, Kars (Eastern Anatolia); and Sanliurfa and Sirnak (Southeastern Anatolia) (Fig. 1). Adult ticks attached to cattle (*Bos taurus*), sheep (*Ovis aries*), dogs (*Canis familiaris*), goats (*Capra aegagrus hircus*), and tortoises (*Testudo graeca*) were removed, kept in separate vials, transferred to the laboratory in dry ice, and identified to species morphologically, using available taxonomic keys [11, 12]. The specimens were pooled according to collection site, sex, host, and species, up to a maximum of 18 individuals, and subsequently were ground by vortexing with tungsten carbide or steel beads (QIAgen, Hilden, Germany), in 200–500 µL of Dulbecco’s phosphate-buffered saline, supplemented with 1% L-glutamine and 5% fetal bovine serum. Following centrifugation at 3000 rpm at 4°C for 6 min, the supernatant from each pool was collected and stored at −80°C. The plasma samples were collected in Antalya (Mediterranean) and Sanliurfa (Southeastern Anatolia) from cattle, sheep, and goats during routine veterinary examinations, and 200 µL of the supernatant or the plasma sample were used for nucleic acid purification using High Pure Viral Nucleic Acid Kit (Roche Diagnostics, Germany), with subsequent complementary DNA synthesis with random hexamers with RevertAid First Strand cDNA Synthesis Kit (Thermo Fisher Scientific, Henningsdorf, Germany), as recommended by the manufacturers. Sample type and tick species distributions according to the region of collection are provided in Table S1.

### Generic amplification of nairoviruses

A singleplex polymerase chain reaction (PCR) amplification assay, targeting viral polymerase central motif A on the nairovirus L genomic segment, was used for screening [13]. Additional information on screening assays is available in Table S2. Amplification mix consisted of 2 mM MgCl_2_, 0.3 mM each dNTP, 0.5 µM forward and reverse primers, 2.5 units of Invitrogen Platinum TaqDNAPolymerase (Thermo Fisher Scientific), and 3 µl of cDNA template in a 30 µl mix. Thermal cycling consisted of an initial denaturation step at 94°C (5 min), followed by 40 cycles of denaturation at 94°C (60 s), gradual increase of 0.2°C/cycle from 42°C for primer binding (60 s) and elongation at 72°C (60 s), with a final elongation at 72°C (3 min). Assay optimization was carried out using CCHFV strain Ank-2 (GenBank accession: MK309333) propagated in SW-13 cells.

### Targeted amplification of JMTV, TcTV1, TcTV2, and TAMV

Four species-specific PCRs were further performed to detect viruses in the samples. We set up nested reactions to amplify NS5-like protein/replicase on segment 1 (JMTV) and nucleoprotein on the S segment (TcTV-1 and TcTV-2), utilizing previously published primers sets [14–16] (Table S2). The reaction mix described for generic nairovirus amplification was used in each reaction. Thermal cycling for the first-round amplifications comprised an initial denaturation at 94°C (4 min), followed by 40 cycles at 94°C (45 s), annealing at 54°C (JMTV), 53°C (TcTVs) (60 s), and 72°C (60 s), with a final extension at 72°C (10 min). Second-round PCRs followed the same conditions except for primer annealing (53°C for JMTV and 52°C for TcTVs). For specific detection of Tamdy virus (TAMV), we used a singleplex PCR to amplify a different section of the RNA-dependent RNA polymerase coding region on the L segment [17] (Table S2), with an identical reaction mix as the generic nairovirus PCR and an annealing temperature of 60°C. TAMV isolate TT1 (NC078320-2), JMTV isolates T14-T15 (MN486261-2), and previously identified TcTV-1 and TcTV-2 positive tick samples were used for assay optimizations [18–20]. Nucleic acid extractions and preparation of amplification mixes were carried out in biosafety class II class cabinets. All standard precautions to prevent carry-over contamination were followed, with extraction, pre-, and post-PCR steps being strictly performed in spatially separated areas. All reaction batches were performed using several non-template controls.

### Amplicon visualization, sequencing, and phylogenetic analysis

Amplified products were visualized in a ChemiDoc XRS + imaging system (Bio-Rad Laboratories, Munich, Germany), following electrophoresis in 1−1.7% agarose gels and stained with SYBR Safe DNA gel stain (Thermo Fisher Scientific, Germany). PCR products of expected size were purified using a commercial kit (GeneJET; ThermoFisher Scientific) according to the manufacturer’s instructions and sequenced in an ABI PRISM 3500xL Dx Genetic Analyzer (Thermo Fisher Scientific). Sequences were handled using Geneious Prime (v2025.0.3) (Biomatters Ltd., Auckland, New Zealand). Minimap2 and CLUSTALW were used for contig mapping, alignment, and pairwise comparisons [21, 22]. BLASTN algorithm was used for similarity searches in the National Center for Biotechnology Information (NCBI) database [23]. Phylogenetic relationships between virus contigs and near relatives were explored using maximum likelihood analysis performed in MEGA v12.0.10 [24]. The optimal model for the phylogenetic and molecular evolutionary analyses in MEGA was determined using the built-in “Find Best DNA/protein-substitution model” tools.

## Results

A total of 1358 samples, comprising 1265 ticks in 192 pools and 93 animal plasma were screened. The ticks were identified as 11 species, including *Haemaphysalis parva* (21.1%), *Rhipicephalus turanicus* (20.2%), *Hyalomma aegyptium* (11.3%), and others (Table S3). Viruses were detected in 36 tick pools (18.7%) and a single plasma sample (1/93, 1.1%). Known tick-borne pathogens were identified in 33 samples (17.2%), comprising 32 ticks pools and a cattle plasma. No co-detection of viral targets or TcTV-2 was observed in any sample. Virus detection was not noted in pools with *Hyalomma anatolicum*, *Ixodes ricinus*, or *Rhipicephalus sanguineus* sensu lato ticks. Information on the samples with virus detection is provided in Table S4.

### Crimean–Congo hemorrhagic fever virus (CCHFV) diversity

**Figure 2:**
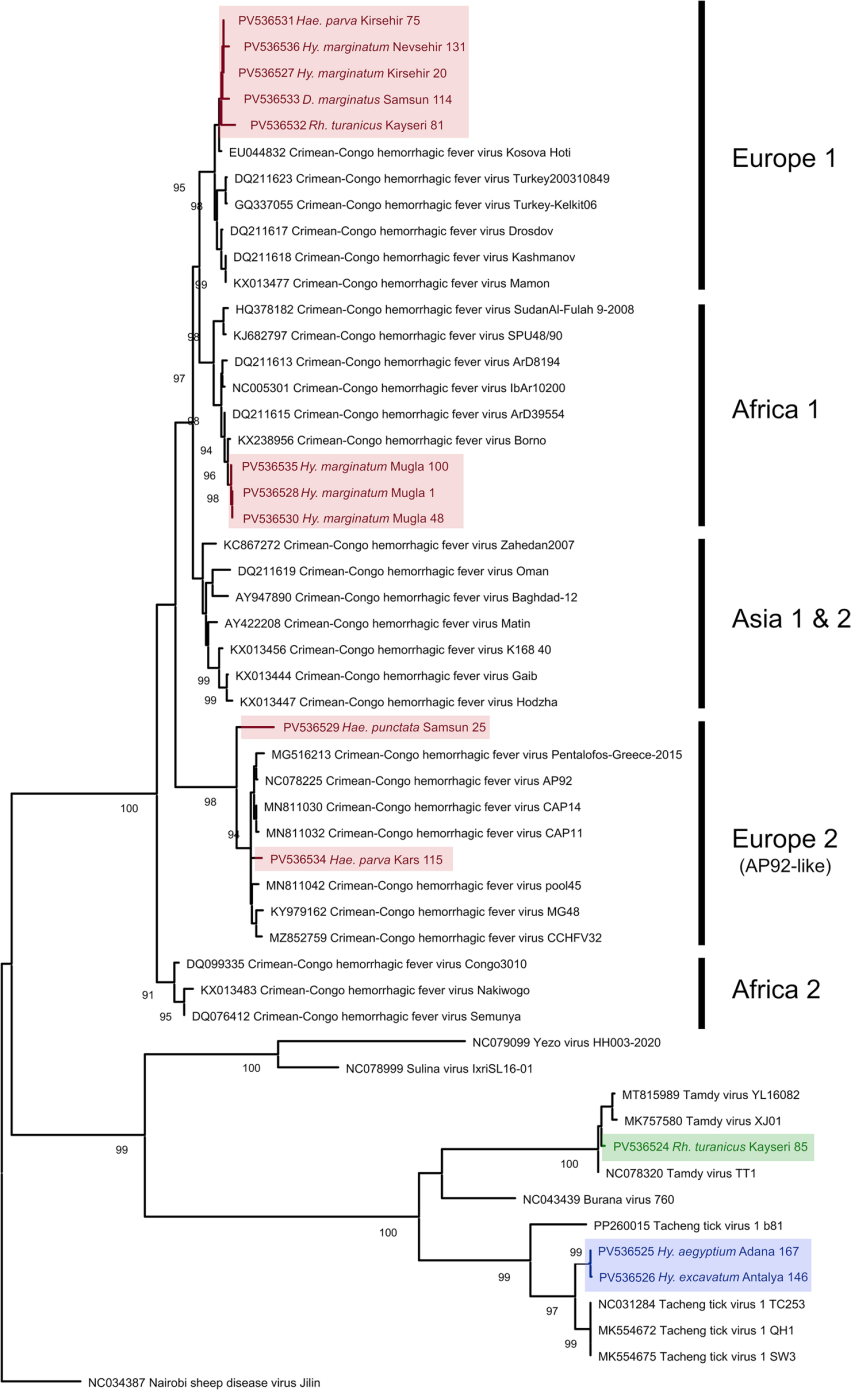
Maximum likelihood tree of Crimean–Congo hemorrhagic fever virus replicase (483 nucleotides), constructed using Tamura 3-parameter model with a discrete Gamma distribution (G) for 500 replications. Sequences generated in the study are color labeled and indicated with GenBank accession, tick species, location, and sample identifier. Bootstrap values lower than 70 are not shown. Virus strains are indicated by GenBank accession and isolate names. Nairobi sheep disease virus isolate Jillin serves as an outgroup

We detected CCHFV sequences produced by generic nairovirus amplification in ten pools with *Hyalomma marginatum* (*n* = 5), *Haemaphysalis parva* (*n* = 2), *Hae. punctata* (*n* = 1), *Dermacentor marginatus* (*n* = 1), and *Rh. turanicus* (*n* = 1) samples (Table S4). Positive pools originated in Central Anatolian provinces (Kayseri, Kirsehir, Nevsehir, *n* = 4), the Aegean province of Mugla (*n* = 3), the Black Sea province of Samsun (*n* = 2), and the Eastern Anatolian province of Kars (*n* = 1). Detection prevalence was highest in *H. marginatum* ticks, with 41.6% (5/12) of the pools observed as positive. Alignment and pairwise comparisons revealed a maximum sequence divergence of 2% (Table S5). In the maximum likelihood tree, we observed that the CCHFV sequences were dispersed among three distinct virus clusters (Fig. 2). While all Central Anatolian sequences and one Black Sea sequence were grouped within the Europe 1 cluster, all sequences from *Hy. marginatum* pools collected in Mugla were placed within Africa 1. Moreover, two sequences from Samsun and Kars provinces were observed as closely related with the former CCHFV genotype Europe 2, currently classified as a separate species (Aigai virus, *Orthonairovirus parahaemorrhagiae*) within *Nairoviridae* [25]. Interestingly, two tick species from Samsun province were noted to harbor distinct genotypes of CCHFV, indicating local co-circulation of diverse virus genomes.

### Tacheng tick virus 1 (TcTV-1) clades

**Figure 3:**
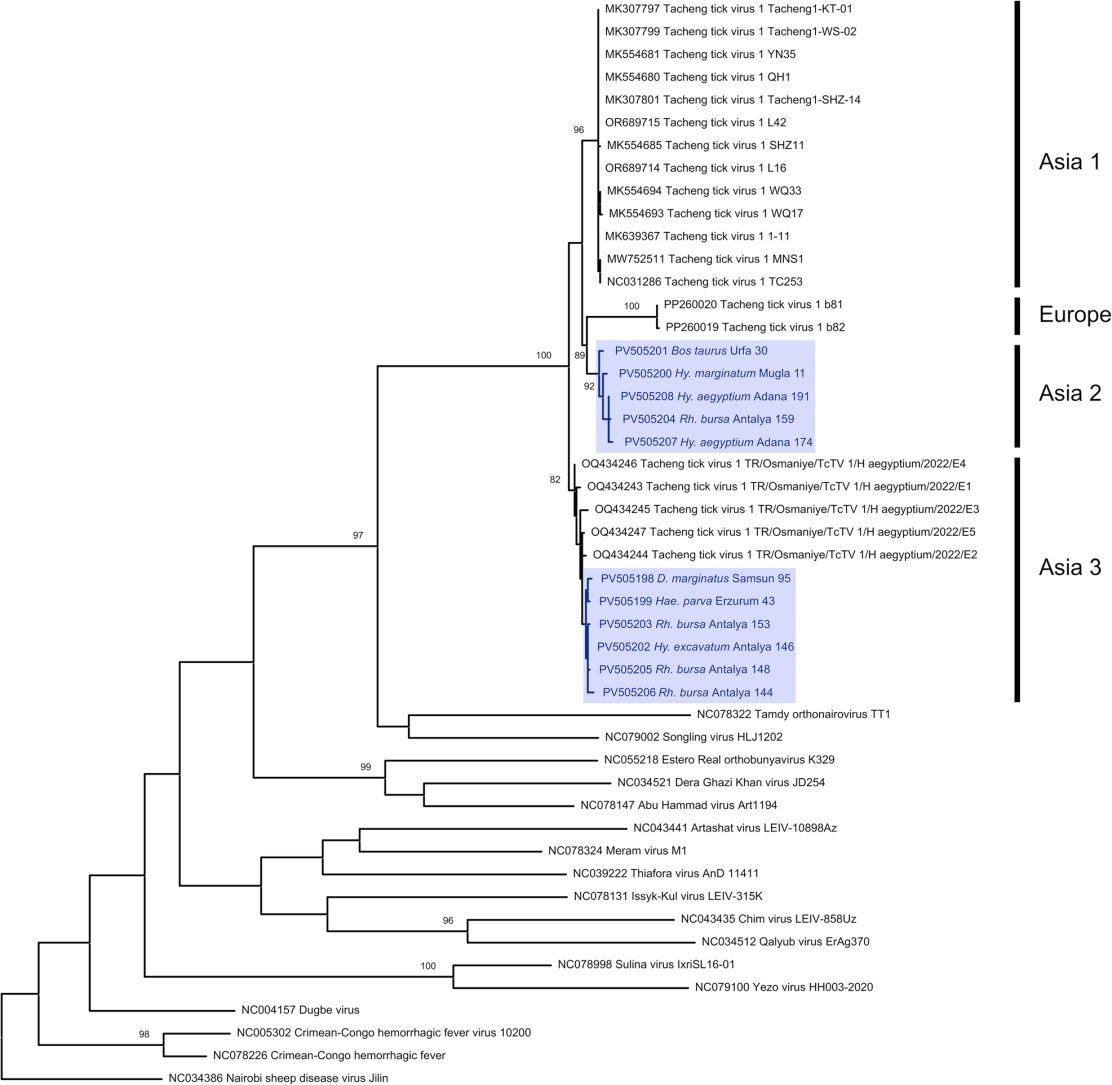
Maximum likelihood tree of Tacheng tick virus 1 nucleoprotein (322 nucleotides) constructed using Tamura 3-parameter model with a discrete Gamma distribution (G) and invariable sites (I) for 500 replications. Sequences generated in the study are color labeled and and indicated with GenBank accession, tick species, location, and sample identifier. Bootstrap values lower than 70 are not shown. Virus strains are indicated by GenBank accession and isolate names. Nairobi sheep disease virus isolate Jillin serves as an outgroup

The most prevalent pathogen observed in the cohort was TcTV-1, detected in 12 tick pools (6.3%) and a cattle plasma (1.1%). Tick species observed to harbor TcTV-1 were *Rhipicephalus bursa* (*n* = 5), *Hy. aegyptium* (*n* = 3), *D. marginatus* (*n* = 1), *Hae. parva* (*n* = 1), *Hy. excavatum* (*n* = 1), and *Hy. marginatum* (*n* = 1). A total of 11 samples including the cattle plasma originating from Sanliurfa province were detected by virus specific amplification, whereas the generic nairovirus amplification yielded two positive samples with one *Hy. excavatum* pool (Antalya146) being reactive in both assays. Pairwise sequence comparisons revealed maximum divergences of 7.9% and 0.3% on the nucleocapsid (Table S5) and polymerase encoding amplicons, respectively. Maximum likelihood analysis based on the partial nucleocapsid sequences showed clustering of global virus genomes into four clades, three originating from Asia (Asia I-III) and one from Europe (I) (Fig. 3). Here, TcTV-1 sequences reported from China and Poland comprise two separate clades, while sequences previously identified from Anatolia including those generated in this study were observed to form two distinct clades, regardless of the infected tick species and location. We also observed a comparable pattern on the trees on the basis of the partial polymerase sequences (Fig. 2), despite having only a single clade from Anatolia being represented. TcTV-1-infected ticks were removed from all screened host species in the study (Table 1).

**Table 1:** Virus detection rates according to tick pools and host species

Tick pool	CCHFV	JMTV	TcTV-1	TAMV	*Nairovirus* sp.
*D. marginatus* (n=20)	1	1	1	0	0
*Hy. aegyptium* (n=32)	0	0	3	0	0
*Hy. excavatum* (n=8)	0	0	1	1	0
*Hy. marginatum* (n=12)	5	0	1	0	0
*Hae. parva* (n=38)	2	6	1	0	4
*Hae. punctata* (n=15)	1	0	0	0	0
*Rh. bursa* (n=18)	0	0	5	0	0
*Rh. turanicus* (n=27)	1	0	0	1	0
	10 (5.2%)	7 (3.6%)	12 (6.3%)	2 (1%)	4 (2%)
Host					
*Bos taurus* (n=19)	4	0	3*	0	0
*Ovis aries* (n=115)	6	7	4	2	4
*Canis familiaris* (n=17)	0	0	1	0	0
*Capra hircus* (n=9)	0	0	4	0	0
*Testudo graeca* (n=32)	0	0	2	0	0

(*CCHFV* Crimean–Congo hemorrhagic fever virus, *TcTV-1* Tacheng tick virus 1, *TAMV* Tamdy virus, *JMTV* Jingmen tick virus)

^*^Includes the single TcTV-1 positive plasma sample

### Tamdy virus (TAMV) phylogeny

**Figure 4:**
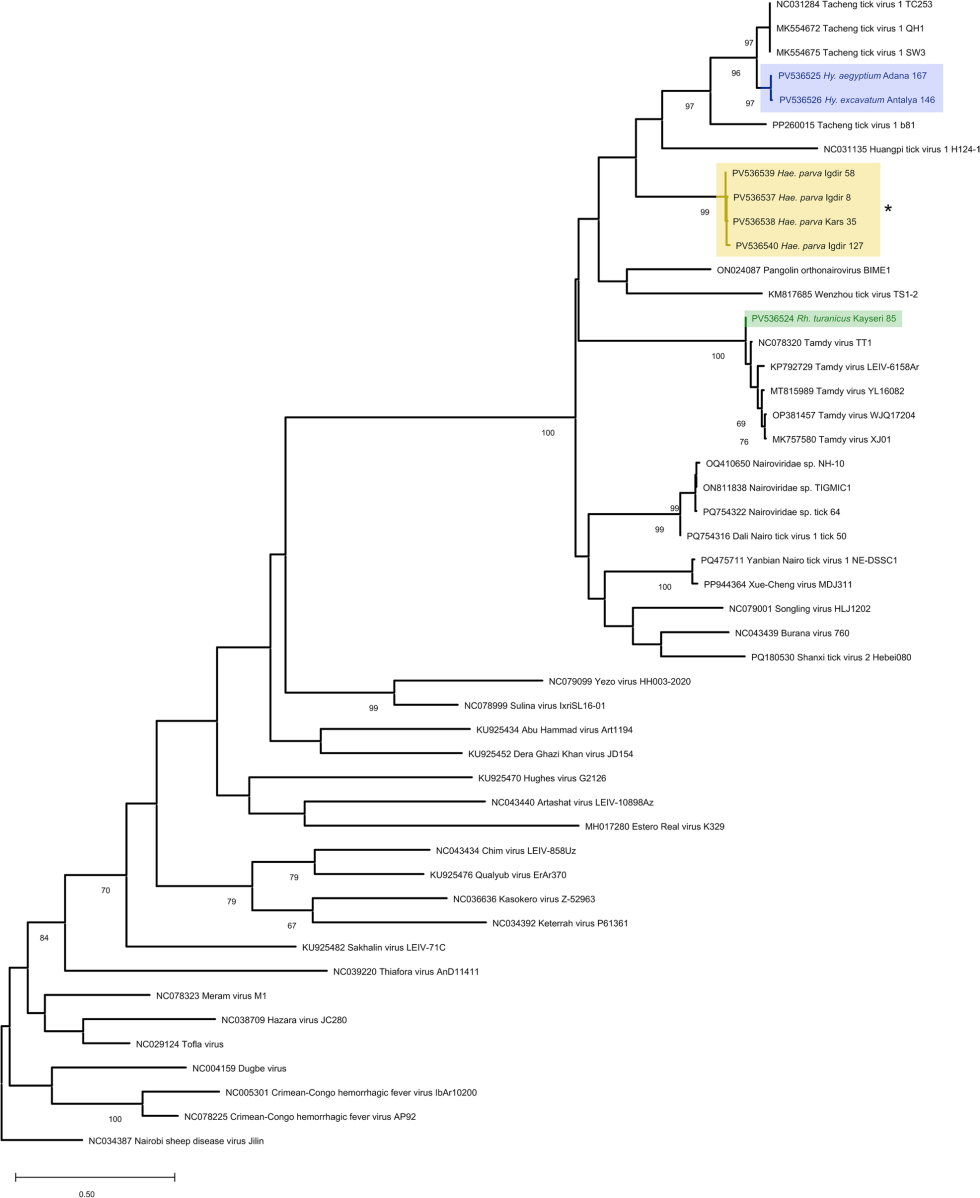
Maximum likelihood tree of nairovirus replicase (342 nucleotides), constructed using Tamura-Nei model with a discrete Gamma distribution (G) for 500 replications. Sequences generated in the study are color labeled and indicated with GenBank accession, tick species, location, and sample identifier. Bootstrap values lower than 70 are not shown. Virus strains are indicated by GenBank accession and isolate names. Baishan forest tick virus serves as an outgroup

Two tick pools from Kayseri province (Central Anatolia) with *Rh. turanicus* and *Hyalomma excavatum* samples were positive for TAMV by specific and generic nairovirus amplification, respectively (Table S4). The maximum likelihood tree of the partial viral replicase amplified by generic nairovirus assay placed the *Rh. turanicus* pool within the TAMV among nairoviruses (Fig. 4). We built a separate tree using the sequence generated by specific amplification in the *Hy. excavatum* pool, which showed grouping with the previously reported TAMV Anatolian virus strain (TT1), among viruses from China and Russia (Fig. S1). Sequence divergence of 1.9% from this strain was noted in pairwise comparisons with both amplicons.

### Jingmen tick virus (JMTV) clustering

**Figure 5:**
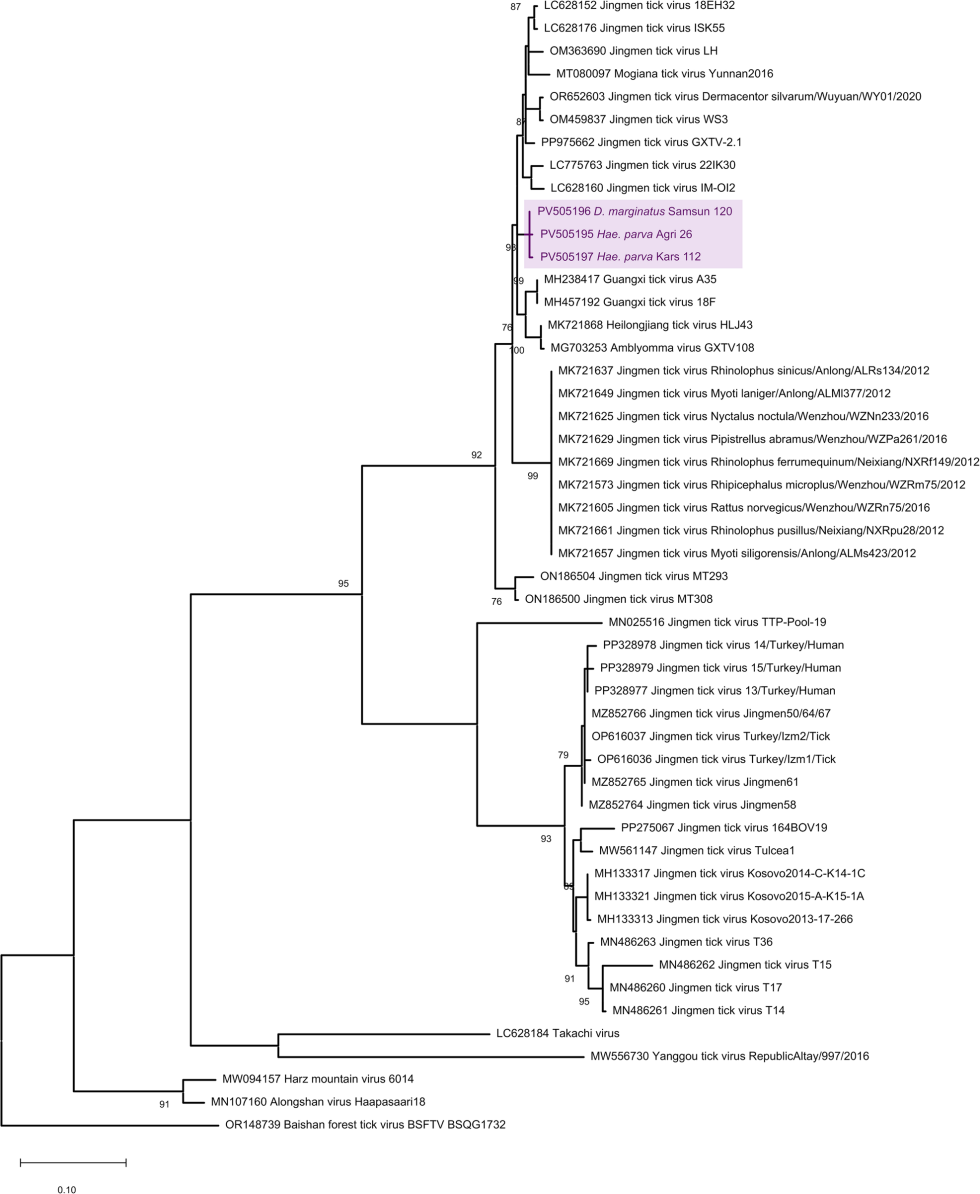
Maximum likelihood tree of Jingmen tick virus NS5-like protein (342 nucleotides) constructed using Tamura-Nei model with a discrete Gamma distribution (G) for 500 replications. Sequence generated in the study is color labeled and indicated with GenBank accession, tick species, location, and sample identifier. Bootstrap values lower than 70 are not shown. Virus strains are indicated by GenBank accession and isolate names. Baishan forest tick virus serves as an outgroup

We generated JMTV amplicons in six *Hae. parva* pools from Eastern Anatolia (Agri and Kars provinces) and one *D. marginatus* pool from the Black Sea province of Samsun, with a total prevalence of 3.6%. Sequences in *Hae. parva* pools were identical, with a low overall divergence (0.4%) in all positive samples (Table S5). In the maximum likelihood analysis, the representative sequences grouped together and remained within the cluster comprising Asian JMTVs and closely related viruses (Fig. 5). Interestingly, complete or partial JMTV genomes previously documented in other regions of Asia Minor formed two distinct clusters, distant to those identified in this study, suggesting multiple JMTV genotypes present in various regions.

### Description of a novel nairovirus

We observed amplification by the generic nairovirus assay in four *Hae. parva* pools from Eastern Anatolia (Igdir and Kars provinces). Sequences generated in these pools showed 98.6–99.3% identities, producing no hits in MEGABLAST searches and outputs with 75% ≥ similarity to various nairoviruses in BLASTN. In the maximum likelihood tree, they formed a well-supported separate cluster, sharing ancestors with TcTV-1 clades within the TAMV genogroup [26] (Fig. 4). Pairwise comparisons with the TcTV-1 and TAMV sequences generated in this study displayed up to 31.6% divergence. We propose these sequences to represent a novel nairovirus belonging in the TAMV genogroup, distantly related to TcTV-1.

## Discussion

The purpose of this cross-sectional study was to investigate tick-borne viral pathogens and provide an update on recently documented viruses from across Anatolia, encompassing 19 provinces from 7 distinct geographical regions. Animal plasma and host-removed ticks, 1358 in total, were collected and screened using generic and specific amplification assays, followed by sequencing. We identified four viral pathogens comprising CCHFV, JMTV, TcTV-1, and TAMV, and a tentative novel nairovirus in 18.7% of the pooled tick and 1.1% of the plasma samples.

We detected CCHFV sequences in 5.2% of the pools spanning five tick genera, including the well-known vector *Hy. marginatum*. CCHFV is the endemic tick-borne virus and the major tick-associated public health threat in Anatolia, with more than 11,000 documented human infections with an average mortality of 4.8% between 2002 and 2018, following the initial emergence of clinical cases [10, 27]. Infections due to CCHFV are widespread in areas of Africa, Eastern Europe, the Middle East, and Asia, mirroring the distribution of the principal *Hyalomma* spp. tick vector [28]. Moreover, CCHFV has recently become a public health concern in Europe, with more than ten countries reporting virus in ticks, vertebrate exposure, or clinical cases, and recent emergence noted in Bulgaria, Greece, Spain, and Portugal [28]. Although widely distributed across Anatolia, the majority of infections have been reported from central and eastern plateaus, especially around the Kelkit Valley [10, 27]. Likewise, the majority of the CCHFV-positive pools in this study originated from central-eastern Anatolian provinces. Interestingly, we observed three distinct CCHFV lineages in the maximum likelihood analyses, including previously reported Europe 1 and 2, as well as Africa 1 [10]. CCHFV isolates have been observed to exhibit significant sequence diversity and are phylogenetically clustered into lineages or clades, mostly overlapping with continental virus distribution [29]. In Anatolia, Europe 1 has been the most frequently documented lineage in ticks and symptomatic humans [10, 30]. Moreover, Europe 2 lineage, which has been reclassified as a separate species [25], has been identified in several tick species from Thrace and Anatolia, at times co-circulating with Europe 1 lineage viruses [18, 19, 31]. However, there are no records of CCHFV’s African lineage documented in humans or ticks in Anatolia. Interestingly, all positive pools comprised *Hy. marginatum* ticks and were collected at the Aegean province of Mugla, where symptomatic cases have been reported. These findings indicate that CCHFV genome diversity in Anatolia is expanding, presumably due to ongoing introductions of divergent virus clades, and potentially as a result of vector carryover by bird migration over the African-Western Palearctic flyway, as previously suggested for Spain [32, 33]. Currently, the impact of newly introduced virus genotypes on virulence or local disease epidemiology is hard to assess. However, it is likely to contribute further to the existing virus diversity via recombination [30], for which *in silico* evidence was previously documented [18]. Hence, the expansion of new and emerging CCHFV lineages in Anatolia require close monitorization.

In the study, TcTV-1 was observed as the most frequently identified tick-borne pathogen, identified in 6 of the 11 species of tick species, with an overall prevalence of 6.3%. Virus genomes were further detected in a cattle plasma from Sanliurfa province (southeastern Anatolia). TcTV-1 is among the newly emerging tick-borne nairoviruses, producing tick-bite-associated febrile disease and skin rash in affected individuals, reported only from China thus far [14]. It was also documented as a co-infecting agent with *Rickettsia* in a case with febrile disease and meningitis, suggesting probable central nervous system (CNS) involvement during infection [34]. In symptomatic cases, virus genomes are present in throat swabs and urine, indicating possible transmission by direct contact with body fluids, as observed in CCHFV. Currently, TcTV-1 has only been reported to circulate in China, Poland, and Türkiye [8, 14, 20, 35]. The main vector of TcTV-1 was initially considered as *Dermacentor* spp.; however, as more information accumulated from various regions, a wider range of susceptible ticks, including several *Hyalomma* spp., were noted [8, 14, 20, 35]. In this study, we further documented TcTV-1 in *Rh. bursa* and *Hae. parva*, adding these tick species to the list of potential vectors. Particular domestic and wild animals were considered as susceptible, with exposure documented in sheep, cattle, and great gerbils (*Rhombomys opimus*) in China [36]. Here, we report virus sequences in cattle plasma, confirming exposure in this species and identifying cattle as a candidate virus reservoir for zoonotic infections. Our detection of infected ticks removed from sheep, goat, and dogs implicate a broader list of domestic animals possibly exposed and potentially participating in circulation. These preliminary findings require verification in larger cohorts to accurately describe TcTV-1 animal reservoirs. Of particular note is the marked TcTV-1 sequence diversity identified in the study. In the maximum likelihood analysis, we observed distinct virus clades corresponding to geographical location (Europe and Asia), as well as within Anatolia, regardless of tick species. These findings suggest local adaptation in virus genomes and possible multiple introductions, likely to impact transmission and virulence. Further investigations on complete TcTV-1 genomes are likely to provide better insights on sequence heterogeneity and impact on vertebrate infections.

Another nairovirus identified in the study is TAMV, with two pools of *Rh. turanicus* and *Hy. excavatum* samples with detectable virus genomes, clustering with TAMV Anatolian strain in the the maximum likelihood analyses. Initially isolated from *Hy. asiaticum* ticks parasitizing sheep in the Tamdinsky district of Uzbekistan in 1971 [37], TAMV was subsequently reported in Uzbekistan, Turkmenistan, Kyrgyzstan, Kazakhstan, Armenia, Azerbaijan, and recently from China [38, 39]. TAMV infections in humans and animals remain understudied, where the virus has historically been associated with sporadic cases of febrile diseases in Kyrgyzstan [38], and human exposure in China and Pakistan [40, 41]. Virus isolation from Bactrian camels and evidence for spillover from ticks to sheep, dogs, and rodents support possible animal reservoirs in nature [39–42]. Recently, various mouse infection models were established to facilitate investigations in pathogenicity [43]. In Anatolia, we initially reported TAMV in a *Hyalomma* spp. pool removed from *Meriones tristrami* (the rodent Tristram’s jird), and subsequently generated the prototype virus complete genome from a *Hy. aegyptium* pool, both collected from Central Anatolia [18, 44]. This region appears as a hotbed zone for TAMV circulation, and *Rhipicephalus* ticks could further be involved in maintenance. Virus replication across different tick species may contribute to previously identified recombinations in local TAMV genomes [18]. Deeper screening in tick populations and investigation of TAMV in cases with febrile disease will help to elucidate TAMV activity in the region and understand risks for further spillover in Asia Minor.

We further detected JMTV with an overall prevalence of 3.6%, mainly from the Eastern Anatolia and Black Sea regions. Although the partial sequences generated in the study lacked considerable diversity, they grouped as a separate clade within the JMTV and JMTV-like virus genomes reported from Asia, distant from JMTVs previously documented in various parts of Anatolia and Thrace [19, 20, 45]. They were detected in *Rhipicephalus*, *Haemaphysalis*, and *Hyalomma* spp. ticks, as well as in bat-collected *Ixodes simplex*. Complete and partial JMTV genomes were observed to form two distinct but related clades, further related with viruses reported from Balkans, with preliminary evidence of recombination [19, 20, 45]. Here, we demonstrate that the JMTV diversity is even more pronounced in Anatolia, with the reporting of this additional virus clade closer to viruses of Asian origin than local strains. JMTV is a globally distributed virus, documented from continental Asia, Africa, Europe Oceania, and North and South America in arthropods, reptiles, and mammals, including cattle, sheep, goats, and horses, in addition to cases with febrile disease [7]. Considerable genome diversity and potential for genomic exchange was noted, with several proposed virus lineages [46]. Interestingly, a recent analysis of JMTV phylogeography revealed a latitude-dependent evolutionary pattern and described three major virus lineages correlated with latitude, frequent intralineage recombination, and global migration events [47]. This might partially explain our findings, where the JMTV positive samples mainly originated from Eastern Anatolian provinces with considerably higher latitudes compared with Mediterranean or Aegean sites. Nevertheless, near-complete genome information will help to understand the extent of JMTV diversity in Anatolia. As JMTV was detected from CCHFV-infected individuals and a proposed impact on disease outcome [48], it is likely to identify similar cases in a CCHFV-endemic region such as Anatolia, for which efforts are ongoing by our group.

Finally, we generated a partial RNA-dependent RNA polymerase sequence of a cryptic nairovirus, highly divergent from its closest relatives in pairwise comparisons and forming a distinct group, sharing ancestors with TcTV-1, TAMV, and pangolin/tick-associated nairoviruses. The cryptic sequence was consistently present in *Hae. parva* pools from Eastern Anatolia and tentatively represents a novel nairovirus, distantly related to TcTV-1. We have previously characterized another novel nairovirus, named Meram virus, in *Hy. aegyptium* ticks from Central Anatolia [18]. Nevertheless, the current virus is only distantly-related to Meram virus among nairoviruses, and near-complete genome sequences are required for better characterization. Identification of replicative forms and RNA-based sequences of genomic segments would provide further evidence toward a replicating virus and exclude endogenous virus elements in tick genomes. Studies are underway for isolation and transcriptome sequencing.

Overall, our screening approach involving host-collected ticks and plasma from potential hosts can be considered a limitation to assessing temporal and spatial patterns virus circulation in locations with detection. Nevertheless, it produced usable information relevant for public health and may be utilized to direct further screening on selected target pathogens in larger cohort sizes. Follow-up studies with human samples and serological testing will enhance the One-Health-based investigations toward these viruses.

## Conclusions

We described a previously undocumented diversity of tick-borne viral pathogens, CCHFV, TcTV-1, and JMTV, in Anatolia. Evidence for probable animal reservoirs of TcTV-1 were further revealed, along with preliminary reporting of a novel nairovirus. These pathogens and TAMV should be considered in the diagnostic workup of cases with symptoms associated with tick bites and in future surveillance efforts.

## Supplementary Information

Supplementary file 1. Figure S1: Maximum likelihood tree of Tamdy virus replicase (420 nucleotides) constructed using Hasegawa-Kishino-Yano model with a discrete Gamma distribution (G) and invariable sites (I) for 500 replications. Sequence generated in the study is color labeled and indicated with GenBank accession, tick species, location, and sample identifier. Bootstrap values lower than 70 are not shown. Virus strains are indicated by GenBank accession and isolate names. Nairobi sheep disease virus isolate Jillin serves as an outgroup

Supplementary file 2. Table S1: Collection locations and distribution of samples in the study. Table S2: Information on amplification assays used in screening. Table S3: Distribution of tick samples according to species. Table S4: Information on tick pools with virus detection.Table S5: Pairwise comparisons of the aligned CCHFV, TcTV-1 (nucleocapsid), and JMTV sequences, expressed as per cent identity

## Data Availability

Sequence data generated in this study are in GenBank under the accessions: PV536528, PV536530, PV536535, PV536527, PV536531, PV536536, PV536532, PV536529, PV536533, PV536534, PV505195, PV505196, PV505197, PV505198, PV505199, PV505202, PV505203, PV505204, PV505205, PV505206, PV505207, PV505208, PV505200, PV505199, PV505198, PV536524, PV536523, PV536537, PV536538, PV536539, and PV536540.

## Abbreviations

CCHFV: Crimean–Congo hemorrhagic fever virus
JMTV: Jingmen tick virus
ORF: Open reading frame
PCR: Polymerase chain reaction
RdRP: RNA-directed RNA polymerase
TcTV-1: Tacheng tick virus 1
TcTV-2: Tacheng tick virus 2
TAMV: Tamdy virus
TBEV: Tick-borne encephalitis virus
