# Supplementary figures and images for: Striking tick-borne virus diversity and potential reservoirs documented during One-Health-based cross-sectional screening in Anatolia
Source: Parasit Vectors. 2025 Oct 10;18:405. doi: 10.1186/s13071-025-07046-w (PMC12512584; doi:10.1186/s13071-025-07046-w)

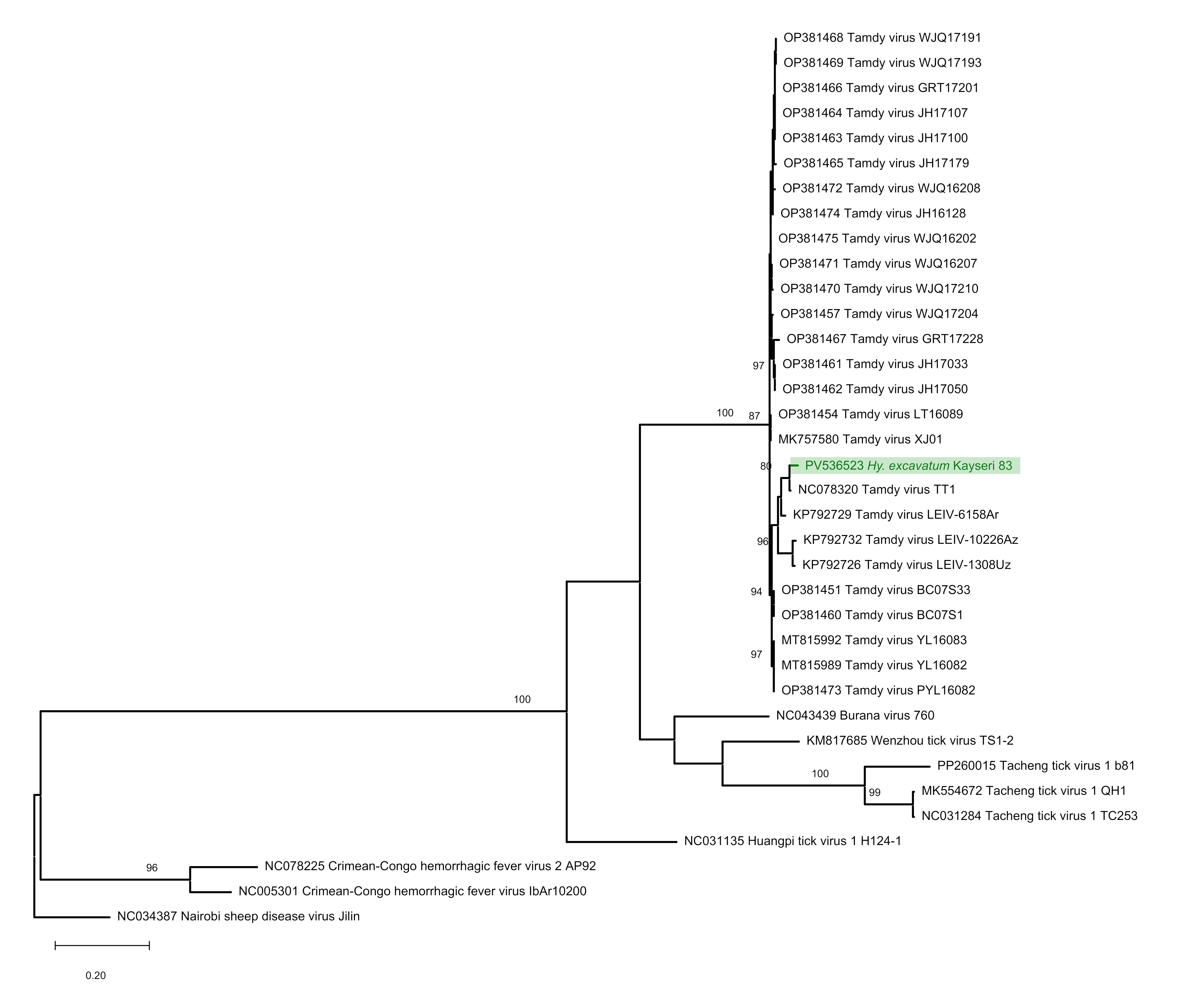

Supplement: Supplementary file 1 — Figure S1: Maximum likelihood tree of Tamdy virus replicase (420 nucleotides) constructed using Hasegawa-Kishino-Yano model with a discrete Gamma distribution (G) and invariable sites (I) for 500 replications. Sequence generated in the study is color labeled and indicated with GenBank accession, tick species, location, and sample identifier. Bootstrap values lower than 70 are not shown. Virus strains are indicated by GenBank accession and isolate names. Nairobi sheep disease virus isolate Jillin serves as an outgroup [file 13071_2025_7046_MOESM1_ESM.jpg]
